# Time-Delay Characteristics of Complex Lü System and Its Application in Speech Communication

**DOI:** 10.3390/e22111260

**Published:** 2020-11-05

**Authors:** Junmei Guo, Chunrui Ma, Zuoxun Wang, Fangfang Zhang

**Affiliations:** 1 School of Electrical Engineering and Automation, Qilu University of Technology (Shandong Academy of Sciences), Jinan 250101, China; gjm@qlu.edu.cn (J.G.); 1043119275@stu.qlu.edu.cn (C.M.); 1043119274@stu.qlu.edu.cn (Z.W.); 2 Shandong Computer Science Center (National Supercomputer Center in Jinan), Shandong Artificial Intelligence Institute, Qilu University of Technology (Shandong Academy of Sciences), Jinan 250101, China

**Keywords:** complex Lü system, controller, self-time-delay synchronization, speech communication

## Abstract

Although complex Lü systems have been considered in many studies, application of the self-time-delay synchronization (STDS) of complex Lü systems in secure speech communications does not appear to have been covered in much of the literature. Therefore, it is meaningful to study the STDS of complex Lü systems and its application in secure speech communication. First, a complex Lü system with double time-delay is introduced and its chaotic characteristics are analyzed. Second, a synchronization controller is designed to achieve STDS. Third, the improved STDS controller is used to design a speech communication scheme based on a complex Lü system. Finally, the effectiveness of the controller and communication scheme are verified by simulation.

## 1. Introduction

The synchronization of complex dynamic chaotic systems can not only reveal many natural phenomena, but also has many applications in image processing, security communication, and mechanical engineering (see [1,2,3,4,5]). Many different synchronization modes have been well-studied, such as combination synchronization, phase synchronization, projection synchronization, and lag synchronization, and many important results have been obtained (see [6,7,8,9,10,11]). A chaotic system with time-delay is better for describing a real physical process than a chaotic system without time-delay. Due to its characteristics, the cipher text formed by a time-delayed chaotic signal exhibits better security. Time-delay chaotic systems are also a hot topic in chaos communication.

Chaos is a definite but unpredictable state of motion [12]. Chaotic systems are unpredictable, controllable, and sensitive to initial values [13]. In 1990, Pecora and Carroll first proposed that chaotic synchronization could be realized by establishing a driver-response system and, on this basis, specific chaotic synchronization circuits could be established [14]. 

In reality, the synchronization of the system is not necessarily simultaneous, and delays can occur. Therefore, a chaotic system may display self-time-delay synchronization (STDS). Time-delay is essential in certain applications. As the time-delay occurs in transmission, it is more suitable to describe the transmitter as the original chaotic system and the receiver as its time-delay system. In fact, it is more practical and economical to consider time-delay for real applications in engineering. In this article, the linear feedback method is used to design a controller to study STDS. 

Many control methods of time-delay chaotic synchronization have been reported [15,16,17,18,19]. A complex Lü system with time-delay has been studied in several papers and in different dimensions [20,21,22], but the application of STDS in secure communication is not present in much of the literature. The STDS controller designed in this article can encrypt by using linear equations for state variables, which is very different from previous encryption methods. The security of the communication scheme for complex chaotic systems is the main problem in speech communication systems. A speech cipher is a solution for transmitting speech information by encrypting data at the sending side and decrypting at the receiving side. Encryption is obtained by scrambling the original spectrum, while decryption is obtained by the reverse process [23,24,25,26]. On the basis of the above discussion, a secure communication scheme was designed. The STDS of a complex Lü chaotic system was used to encrypt the voice. The simulation results show that the communication scheme is very good. The main contributions of this paper are as follows:

1. The time-delay characteristics of complex Lü systems with different parameters are studied and a controller for realizing STDS is designed;
2. The evolved STDS controller is applied to the encryption of speech communication using linear equations of state variables and its effect is verified by simulation experiments.

The rest of this paper is structured as follows: In Section 2, the characteristics of complex Lü systems with time-delays are studied. In Section 3, a synchronous controller is designed and the mathematical proof for establishing self-time-delay synchronous control is given. In Section 4, speech signal transmission encryption is realized through the existing chaotic model of a complex Lü system. In Section 5, simulations are performed to verify the effectiveness of the STDS controller and to implement speech transmission encryption. Finally, the study is summarized in Section 6.

## 2. Characteristics of Complex Lü Chaotic Systems with Time-Delay

The system of equations of a complex Lü system is as follows:

(1) $$\left\{\begin{array}{l} {\dot{y}}_{1}=a_{1}(y_{2}-y_{1})\\ {\dot{y}}_{2}=a_{2}y_{2}-y_{1}y_{3}\\ {\dot{y}}_{3}=-a_{3}y_{3}+({\bar{y}}_{1}y_{2}+y_{1}{\bar{y}}_{2})/2 \end{array}\right.$$

where $y_{1}=u_{1}+ju_{2}$ , $y_{2}=u_{3}+ju_{4}$ are complex variables, $y_{3}=u_{5}$ is the real state variable, and $a_{1},a_{2},a_{3}$ are constants. By separating the real and imaginary parts of the system equation, a five-dimensional chaotic system of equations can be obtained, as follows:

(2) $$\left\{\begin{array}{l} {\dot{u}}_{1}=a_{1}(u_{3}-u_{1})\\ {\dot{u}}_{2}=a_{1}(u_{4}-u_{2})\\ {\dot{u}}_{3}=a_{2}u_{3}-u_{1}u_{5}\\ {\dot{u}}_{4}=a_{2}u_{4}-u_{2}u_{5}\\ {\dot{u}}_{5}=-a_{3}u_{5}+u_{1}u_{3}+u_{2}u_{4} \end{array}\right.$$

Consider the following time-delay complex Lü system of equations:

(3) $$\left\{\begin{array}{l} {\dot{x}}_{1}=a_{1}(x_{2}-x_{1})\\ {\dot{x}}_{2}=-x_{1}x_{3}+a_{2}x_{2}(t-\tau_{1})\\ {\dot{x}}_{3}=-a_{3}x_{3}(t-\tau_{0})+({\bar{x}}_{1}x_{2}+x_{1}{\bar{x}}_{2})/2 \end{array}\right.$$

where $0<\tau_{0,1}\leq \tau_{m}$ is the delay factor, $x_{1}=u_{1}^{\prime }+ju_{2}^{\prime }$ , $x_{2}=u_{3}^{\prime }+ju_{4}^{\prime }$ are the complex variables, and $x_{1}=u_{5}^{\prime }$ is the real state variable. By separating the real and imaginary parts of the system of equations, a five-dimensional time-delay chaotic system of equations can be obtained.

(4) $$\left\{\begin{array}{l} \dot{u_{1}^{\prime }}=a_{1}({u^{\prime }}_{3}-{u^{\prime }}_{1})\\ {\dot{u^{\prime }}}_{2}=a_{1}({u^{\prime }}_{4}-{u^{\prime }}_{2})\\ {\dot{u^{\prime }}}_{3}=a_{2}{u^{\prime }}_{3}(t-\tau_{1})-u_{1}^{\prime }{u^{\prime }}_{5}\\ {\dot{u^{\prime }}}_{4}=a_{2}{u^{\prime }}_{4}(t-\tau_{1})-{u^{\prime }}_{2}{u^{\prime }}_{5}\\ {\dot{u^{\prime }}}_{5}=-a_{3}u_{5}^{\prime }(t-\tau_{0})+u_{1}^{\prime }u_{3}^{\prime }+{u^{\prime }}_{2}{u^{\prime }}_{4} \end{array}\right.$$

### 2.1. The Dissipation

The divergence of a complex chaos system can be expressed as follows:

(5) $$\nabla V=\frac{\partial {\dot{u}}_{1}}{\partial u_{1}}+\frac{\partial {\dot{u}}_{2}}{\partial u_{2}}+\cdot \cdot \cdot +\frac{\partial {\dot{u}}_{i}}{\partial u_{i}}$$

Therefore, the divergence of system (2) can be expressed as $\nabla =-2a_{1}+2a_{2}-a_{3}$, $-2a_{1}+2a_{2}-a_{3}>0$, where the system is dissipative and converges in exponential form, $e^{(-2a_{1}+2a_{2}-a_{3})t}$. In fact, the volume element of the initial volume V(0) becomes $V(t)=V(0)e^{(-2a_{1}+2a_{2}-a_{3})t}$ at time *T*. When t→∞, each small volume element—including the trajectory of system (3)—converges to zero at an exponential rate $e^{(-2a_{1}+2a_{2}-a_{3})t}$ and its motion is fixed on an attractor, which indicates that there exists an attractor in system (3).

### 2.2. Sensitivity of Initial Values and Symmetry

**Figure 1:**
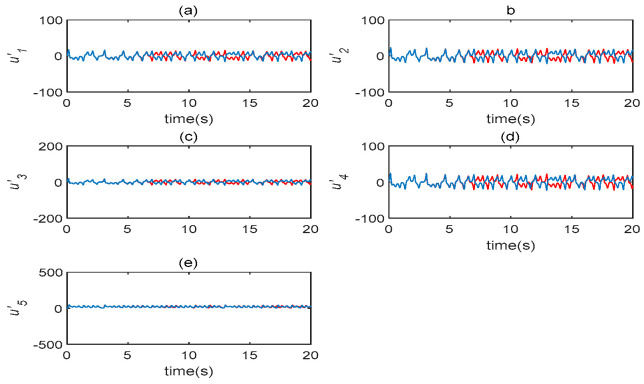
The evolution diagrams of five state variables in the time-delaycomplex Lü system (τ=5s). (**a**) ${u^{\prime }}_{1}$ state changes with time; (**b**) ${u^{\prime }}_{2}$ state changes with time; (**c**) ${u^{\prime }}_{3}$ state changes with time; (**d**) ${u^{\prime }}_{4}$ state changes with time; (**e**) ${u^{\prime }}_{5}$ state changes with time.

In terms of its sensitivity to initial values, a time-delay complex Lü chaotic system can be analyzed. Let τ=5s and select two very similar initial values, such as ${\left(1,2,3,4,1\right)}^{T}$ and ${\left(1.001,2.001,3,4,1.001\right)}^{T}$. The evolution diagrams of five state variables are shown in Figure 1, which demonstrates that the time-delay system is highly sensitive to the initial state value. The following transformation is introduced to system (3): $\left(x_{1},x_{2},x_{3})\to (-x_{1},-x_{2},x_{3}\right)$; under this, system (3) remains unchanged. As a result of this, system (3) is symmetrical with respect to $x_{3}$; this symmetry is true for all parameters.

### 2.3. Chaotic Characteristics under Different Time-Delay Factors

**Figure 2:**
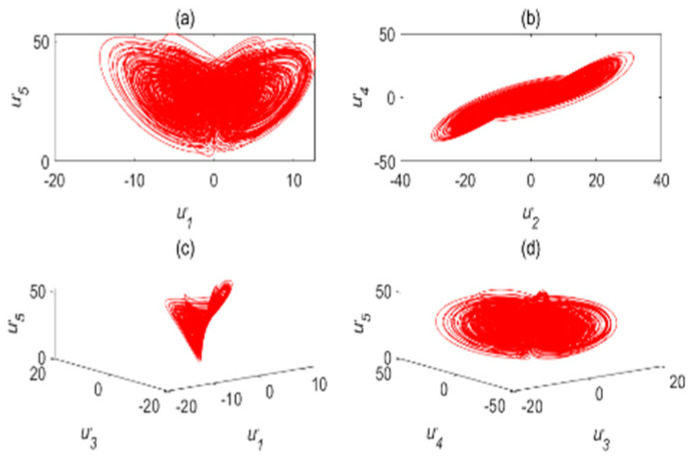
Chaotic attractor phase diagram of a complex Lü system. (**a**) $\left(u_{1},u_{5}\right)$; (**b**) $\left(u_{2},u_{4}\right)$; (**c**) $\left(u_{1},u_{3},u_{5}\right)$; (**d**) $\left(u_{3},u_{4},u_{5}\right)$.

**Figure 3:**
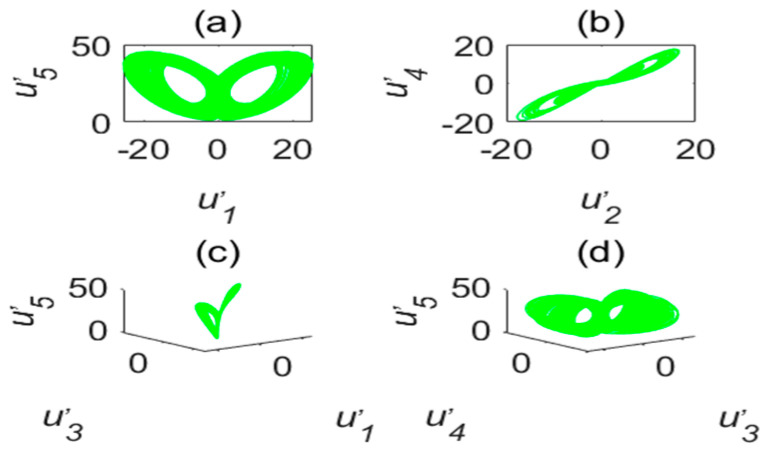
Chaotic attractor phase diagram of the time-delay complex Lü system (τ=17s). (**a**) $\left({u^{\prime }}_{1},{u^{\prime }}_{5}\right)$; (**b**) $\left({u^{\prime }}_{2},{u^{\prime }}_{4}\right)$; (**c**) $\left({u^{\prime }}_{1},{u^{\prime }}_{3},{u^{\prime }}_{5}\right)$; (**d**) $\left({u^{\prime }}_{3},{u^{\prime }}_{4},{u^{\prime }}_{5}\right)$.

**Figure 4:**
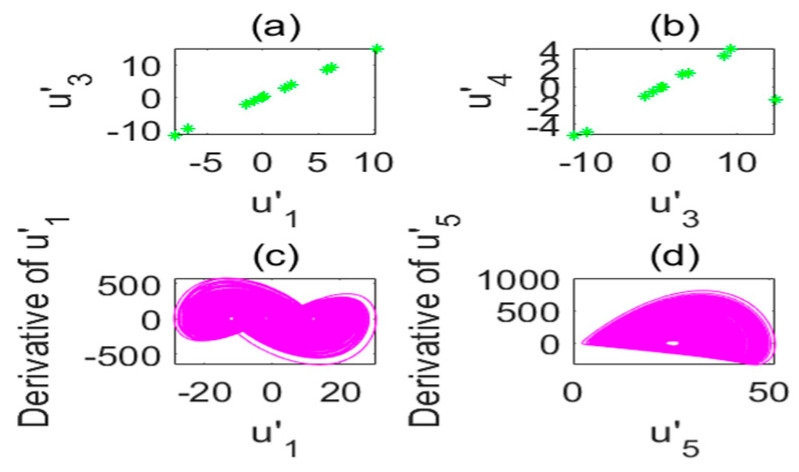
Poincare section diagram and phase diagram of the time-delay complex Lü system (τ=3s). (**a**) $\left({u^{\prime }}_{1},{u^{\prime }}_{3}\right)$; (**b**) $\left({u^{\prime }}_{3},{u^{\prime }}_{4}\right)$; (**c**) cross section of the state variable ${u^{\prime }}_{1}$ and its derivative ${u^{\prime }}_{1}$; (**d**) cross section of the state variable ${u^{\prime }}_{5}$ and its derivative ${u^{\prime }}_{5}$.

**Figure 5:**
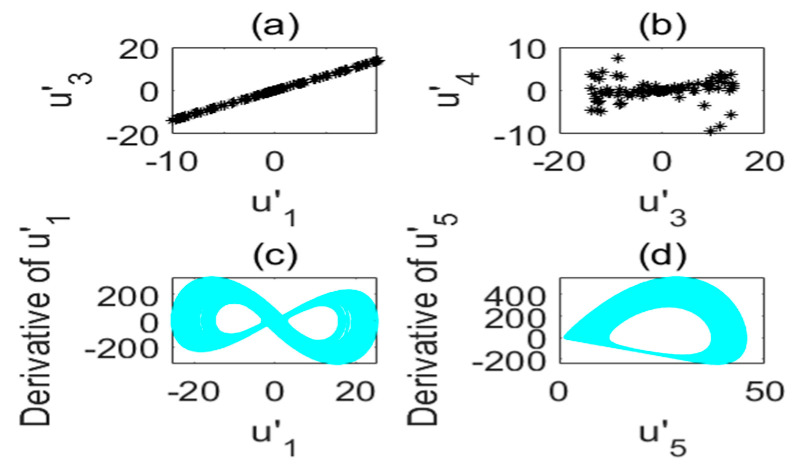
Poincare section diagram and phase diagram of the time-delay complex Lü system (τ=17s). (**a**) $\left({u^{\prime }}_{1},{u^{\prime }}_{3}\right)$; (**b**) $\left({u^{\prime }}_{3},{u^{\prime }}_{4}\right)$; (**c**) cross section of the state variable ${u^{\prime }}_{1}$ and its derivative ${u^{\prime }}_{1}$; (**d**) cross section of the state variable ${u^{\prime }}_{5}$ and its derivative ${u^{\prime }}_{5}$.

The chaotic characteristics of a time-delay complex Lü chaotic system are quite different from those of the original system, as the time-delay system has high randomness and an unpredictable time-series. Figure 2 is chaotic attractor phase diagram of a complex Lü system. When τ=17s, the chaotic attractor phase diagram of the time-delay complex Lü system is shown in Figure 3. Moreover, due to changes in the time-delay parameters of time-delay systems, it can be seen from the Poincare section diagrams (Figure 4 and Figure 5), that time-delay complex Lü systems display great differences under different delay factors.

**Figure 6:**
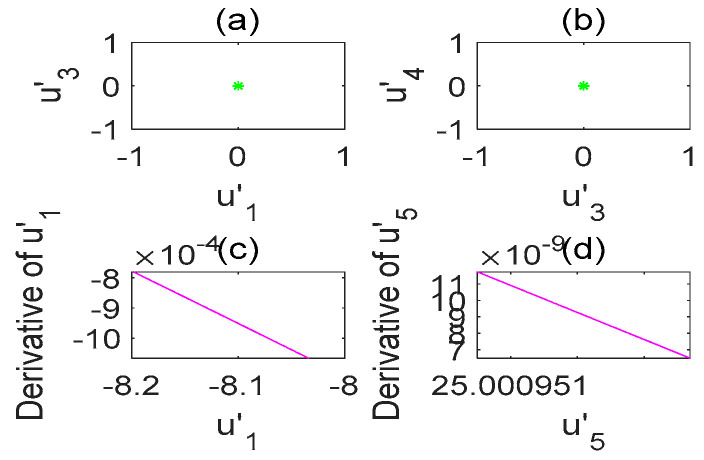
Poincare section diagram and phase diagram of the time-delay complex Lü system ($\tau_{1}=18s$). (**a**) $\left({u^{\prime }}_{1},{u^{\prime }}_{3}\right)$; (**b**) $\left({u^{\prime }}_{3},{u^{\prime }}_{4}\right)$; (**c**) cross section of the state variable ${u^{\prime }}_{1}$ and its derivative ${u^{\prime }}_{1}$; (**d**) cross section of the state variable ${u^{\prime }}_{5}$ and its derivative ${u^{\prime }}_{5}$.

**Figure 7:**
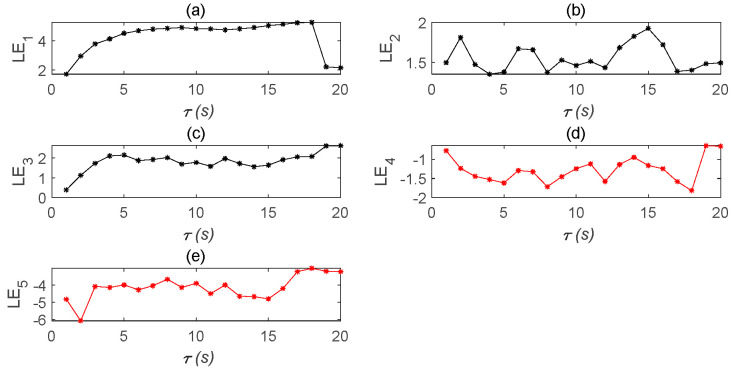
Lyapunov exponent diagram of five state variables of the time-delay complex Lü system. (**a**) Lyapunov exponent diagram of ${u^{\prime }}_{1}$ state variable changing with time; (**b**) Lyapunov exponent diagram of ${u^{\prime }}_{2}$ state variable changing with time; (**c**) Lyapunov exponent diagram of ${u^{\prime }}_{3}$ state variable changing with time; (**d**) Lyapunov exponent diagram of ${u^{\prime }}_{4}$ state variable changing with time; (**e**) Lyapunov exponent diagram of ${u^{\prime }}_{5}$ state variable changing with time.

The stability of a time-delay complex Lü system is also related to the values of $a_{1},a_{2},a_{3},\tau_{1},\tau_{2}$. Therefore, in order to observe the system more clearly, the initial values of $a_{1},a_{2},a_{3}$ must remain unchanged and only the value of τ is changed; however, the time-delay τ still needs to meet the condition 1s≤τ<18s. This is because, according to the definition of the Lyapunov function employed to determine whether the system is in a chaotic state, there needs to be at least one positive Lyapunov exponent. Therefore, it can be judged from Figure 6 and Figure 7 that the chaos disappears when τ=18s.

**Remark** **1.** $\tau_{1}$ *must satisfy the condition* $1s\leq \tau_{1}<18s$ *, in order for the time-delay complex Lü system to be chaotic.*

**Remark** **2.** *In the simulation experiment, with increasing time-delay* τ *, it can be found that the system is no longer in a chaotic state when* $\tau_{1}=18$ *. When* $\tau_{1}$ *is fixed at* $\tau_{1}=17$ *, the value of* $\tau_{0}$ *can continue to increase and the system will still be chaotic.*

### 2.4. Chaotic Characteristics of Time-Delay Complex Lü Systems with Different Parameters

**Figure 8:**
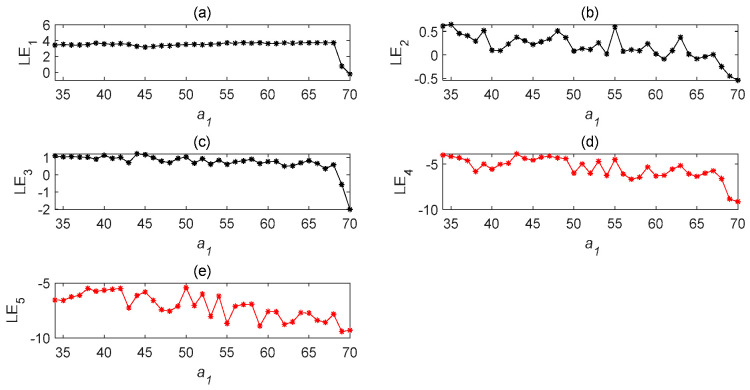
Lyapunov exponent diagram of the time-delay complex Lü system with parameter $a_{1}$. (**a**) Lyapunov exponent diagram of ${u^{\prime }}_{1}$ state variable changing with $a_{1}$; (**b**) Lyapunov exponent diagram of ${u^{\prime }}_{2}$ state variable changing with $a_{1}$; (**c**) Lyapunov exponent diagram of ${u^{\prime }}_{3}$ state variable changing with $a_{1}$; (**d**) Lyapunov exponent diagram of ${u^{\prime }}_{4}$ state variable changing with $a_{1}$; (**e**) Lyapunov exponent diagram of ${u^{\prime }}_{5}$ state variable changing with $a_{1}$ (τ=5s).

**Figure 9:**
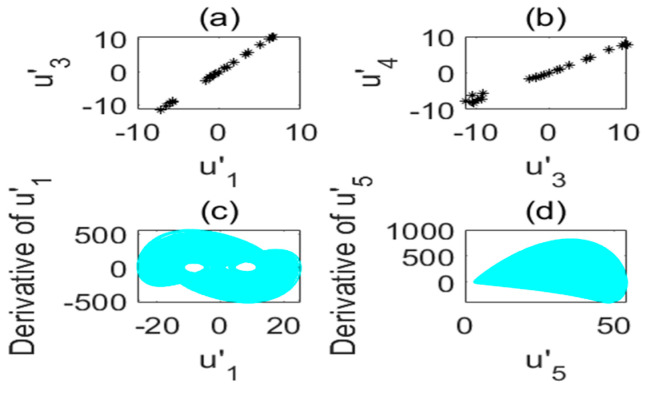
Poincare section diagram and phase diagram of the time-delay complex Lü system. (**a**) $\left({u^{\prime }}_{1},{u^{\prime }}_{3}\right)$; (**b**) $\left({u^{\prime }}_{3},{u^{\prime }}_{4}\right)$; (**c**) cross section of the state variable ${u^{\prime }}_{1}$ and its derivative ${u^{\prime }}_{1}$; (**d**) cross section of the state variable ${u^{\prime }}_{5}$ and its derivative ${u^{\prime }}_{5}$ ($a_{1}=34,\tau =5s$).

**Figure 10:**
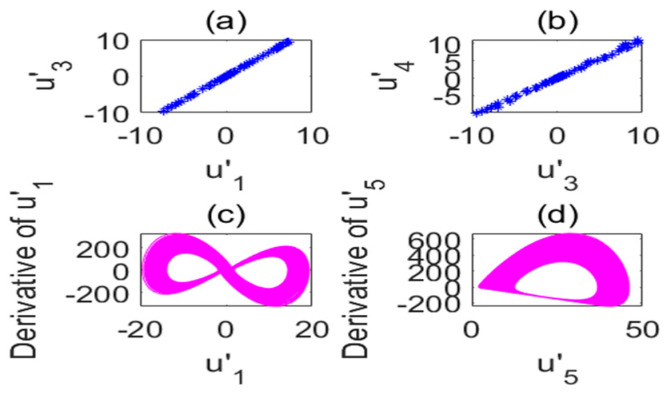
Poincare section diagram and phase diagram of the time-delay complex Lü system. (**a**) $\left({u^{\prime }}_{1},{u^{\prime }}_{3}\right)$; (**b**) $\left({u^{\prime }}_{3},{u^{\prime }}_{4}\right)$; (**c**) cross section of the state variable ${u^{\prime }}_{1}$ and its derivative ${u^{\prime }}_{1}$; (**d**) cross section of the state variable ${u^{\prime }}_{5}$ and its derivative ${u^{\prime }}_{5}$ ($a_{1}=66,\tau =5s$).

Under different parameters, the characteristics of time-delay complex Lü systems are also different. Therefore, we first observe the chaotic characteristics of a time-delay complex Lü system with the change in $a_{1}$. In order to test the influence of the $a_{1}$ parameter, the values of $a_{2},a_{3},\tau_{1},\tau_{2}$ are kept unchanged. It can be seen, from the Lyapunov exponent diagram in Figure 8, that when the $a_{1}$ parameter is within the specified range, the system is chaotic; while the system beyond the value $a_{1}=66$ is not chaotic. When $a_{1}=67$, it can be seen, from Figure 8, that its values are all negative numbers, and so Lyapunov’s definition of judging whether the system is chaotic is not satisfied. In order to observe the image clearly, the initial value was set to $a_{1}=33.5$ and the exponential graph of the Lyapunov function varying with the parameter was developed. After many observations, when the value of $a_{1}$ exceeded 66, the system no longer exhibited chaotic behavior. It can be seen from Figure 9 and Figure 10 that the influences of different parameters of the system on the time-delay complex Lü system are relatively large.

When $a_{1}=67$, the Poincare cross-sectional view of the system is the same as in Figure 6, the chaotic phenomenon of the system disappears, and there is only one point left (figure not shown to avoid repetition). Therefore, it is consistent with the previous conclusion drawn by the Lyapunov exponent chart. Similarly, changes in the other two parameters can also be discussed through numerical simulation.

## 3. STDS of a Complex Lü System

### 3.1. Definition of Self-Delay Synchronization

Original system:

(6) $$\dot{y}(t)=f(y(t)),y(t)={\left\{y_{1},(,t,)\right.,y_{2}(t),\ldots ,\left.y_{n}(t)\right\}}^{T}$$

Time-delay system:

(7) $$\dot{x}(t)=f(x(t-\tau ))+v(x(t-\tau ),x(t),y(t)),\;x(t)={\left\{x_{1}(t)\right.,x_{2}(t),\ldots ,\left.x_{n}(t)\right\}}^{T}$$

where x(t),y(t) represent the complex state variables and $\tau ={\left\{\tau_{1},\tau_{2},\ldots ,\tau_{n}\right\}}^{T}$, $\left(\tau_{i}\geq 0,i=1,2,\ldots ,n\right)$ is the time-delay factor vector. When there exists a controller *v*,

(8) $$\lim{\left\|x(t)-y(t)\right\|}^{2}=\lim{\left\|x{\left(t\right)}^{r}-y{\left(t\right)}^{r}\right\|}^{2}+{\left\|x{\left(t\right)}^{i}-y{\left(t\right)}^{i}\right\|}^{2}=0\left(t\to +\infty \right)$$

where *x(t)* and *y(t)* represent self-time-delay synchronization [27,28,29,30,31].

**Remark** **3.** *If*τ=0, *the STDS is equivalent to complete synchronization. Therefore, STDS includes complete synchronization and further extends complete synchronization.*

### 3.2. Design of the STDS Controller

**Lemma** **1.** *Consider a linear continuous time-delay system*

(9) $$\dot{z}(t)=A(t)z(t)+B_{1}z(t-\tau_{0})+B_{2}z(t-\tau_{1})$$

*where* $z(t)\in R^{n}$ *,* A(t) *is* n×n *time-varying real matrix,* $B_{1},B_{2}$ *are* n×n *constant real matrices,* $\tau_{1},\tau_{0}>0$ *. If there is a positive definite matrix P, M1, M2 satisfying the negative definite matrix (10),*

(10) $$\left(\begin{array}{ccc} A{\left(t\right)}^{T}P+PA(t)+M_{1}+M_{2} & \frac{PB_{1}+{B_{1}}^{T}P}{2} & \frac{PB_{2}+{B_{2}}^{T}P}{2}\\ \frac{PB_{1}+{B_{1}}^{T}P}{2} & -M_{1} & 0\\ \frac{PB_{2}+{B_{2}}^{T}P}{2} & 0 & -M_{2} \end{array}\right)$$

*then z(t) = 0 is the global stability point of the system (9).*

**Theorem** **1.** *If the complex L ü system (2) is used as the main system, the following controlled time-delay complex Lü system is taken as the slave system:*

(11) $$\left\{\begin{array}{l} \dot{u_{1}^{\prime }}=a_{1}({u^{\prime }}_{3}-u_{1}^{\prime })+v_{1}\\ {\dot{u^{\prime }}}_{2}=a_{1}({u^{\prime }}_{4}-{u^{\prime }}_{2})+v_{2}\\ {\dot{u^{\prime }}}_{3}=a_{2}{u^{\prime }}_{3}(t-\tau_{1})-{u^{\prime }}_{1}{u^{\prime }}_{5}+v_{3}\\ {\dot{u^{\prime }}}_{4}=a_{2}{u^{\prime }}_{4}(t-\tau_{1})-{u^{\prime }}_{2}{u^{\prime }}_{5}+v_{4}\\ {\dot{u^{\prime }}}_{5}=-a_{3}{u^{\prime }}_{5}(t-\tau_{0})+u_{1}^{\prime }{u^{\prime }}_{3}+{u^{\prime }}_{2}{u^{\prime }}_{4}+v_{5} \end{array}\right.$$

*the STDS controller is designed as follows:*

(12) $$\left\{\begin{array}{l} v_{1}=k_{1}e_{1}\\ v_{2}=k_{2}e_{2}\\ v_{3}=u_{1}^{\prime }u_{5}^{\prime }-u_{1}u_{5}+k_{3}e_{3}+a_{2}(u_{3}-u_{3}(t-\tau_{1}))\\ v_{4}=u_{2}^{\prime }{u^{\prime }}_{5}-u_{2}u_{5}+k_{4}e_{4}+a_{2}(u_{4}-u_{4}(t-\tau_{1}))\\ v_{5}=a_{3}(-u_{5}+u_{5}(t-\tau_{0}))-u_{1}^{\prime }u_{3}^{\prime }-u_{2}^{\prime }u_{4}^{\prime }+u_{1}u_{3}+u_{2}u_{4}+k_{5}e_{5} \end{array}\right.$$

*where*$e_{i}(t)=u_{i}^{\prime }(t)-u_{i}(t)$*,*$e_{i}(t-\tau )=u_{i}^{\prime }(t-\tau )-u_{i}(t-\tau )$*,*i=1,2,3,4,5*,then there are*$k_{i}\in R,(i=1,2,3,4,5)$*making*$\lim_{t\to +\infty }\sum_{i=1}^{5}{\left(u_{i}^{\prime }-u_{i}\right)}^{2}=0$, *which establishes the STDS between the complex Lü system (2) and the time-delay complex Lü system (11).*

**Proof**  Let $e_{i}(t)=u_{i}^{\prime }(t)-u_{i}(t)$, $e_{i}(t-\tau )=u_{i}^{\prime }(t-\tau )-u_{i}(t-\tau )$. The time-delay error system of equations can then be obtained as

(13) $$\left\{\begin{array}{l} {\dot{e}}_{1}=k_{1}e_{1}+a_{1}(e_{3}-e_{1})\\ {\dot{e}}_{2}=k_{2}e_{2}+a_{1}(e_{4}-e_{2})\\ {\dot{e}}_{3}=a_{2}e_{3}(t-\tau_{1})+\;k_{3}e_{3}\\ {\dot{e}}_{4}=a_{2}e_{4}(t-\tau_{1})+\;k_{4}e_{4}\\ {\dot{e}}_{5}=-a_{3}e_{5}(t-\tau_{0})+\;k_{5}e_{5} \end{array}\right.$$

where $A(t)=(\begin{array}{ccccc} k_{1}-a_{1} & & & & \\ & k_{2}-a_{1} & & & \\ & & k_{3} & & \\ & & & k_{4} & \\ & & & & k_{5} \end{array}),B_{1}=(\begin{array}{ccccc} 0 & & & & \\ & 0 & & & \\ & & 0 & & \\ & & & 0 & \\ & & & & -a_{3} \end{array}),B_{2}=(\begin{array}{ccccc} 0 & & & & \\ & 0 & & & \\ & & a_{2} & & \\ & & & a_{2} & \\ & & & & 0 \end{array})$.

As $u_{i}(i=1,2,3,4,5)$ is a bounded system state variable and $a_{1},a_{2},a_{3}$ are constant, the matrix *A(t)* is bounded. Therefore, system (13) can be regarded as a linear time-varying system. Suppose $P=I,M_{1}=4I,M_{2}=10I$. Equation (10) can then be transformed into

(14) $$\left(\begin{array}{ccc} A^{T}+A+14I & \frac{B_{1}+{B_{1}}^{T}}{2} & \frac{B_{2}+{B_{2}}^{T}}{2}\\ \frac{B_{1}+{B_{1}}^{T}}{2} & -4I & 0\\ \frac{B_{2}+{B_{2}}^{T}}{2} & 0 & -10I \end{array}\right)$$

where $A^{T}(t)+A(t)+14I$= $\left(\begin{array}{ccccc} 2k_{1}‒2a_{1}+14 & & & & \\ & 2k_{2}‒a_{1}+14 & & & \\ & & 2k_{3}+14 & & \\ & & & 2k_{4}+14 & \\ & & & & 2k_{5}+14 \end{array}\right)$

According to Lemma 1, the real symmetric matrix (14) becomes the dominant row diagonal matrix and requires all diagonal elements to be negative. Therefore, the following criteria must be satisfied.

(15) $$\begin{array}{c} \mathrm{Row}\;1:2k_{1}-2a_{1}+14<0;\\ \mathrm{Row}\;2:2k_{2}-2a_{1}+14<0;\\ \mathrm{Row}\;3:\left|2k_{3}+14\right|>\left|a_{2}\right|,2k_{3}+14<0;\\ \mathrm{Row}\;4:\left|2k_{4}+14\right|>\left|a_{2}\right|,2k_{4}+14<0;\\ \mathrm{Row}\;5:\left|2k_{5}+14\right|>\left|a_{3}\right|,2k_{5}+14<0. \end{array}$$

According to the Lyapunov stability theory, $k_{1,2}<a_{1}-7$, $k_{3,4}<-7-\left|a_{2}\right|/2$, and $k_{5}<-7-\left|a_{3}\right|/2$, which make the error system asymptotically stable. The master system (2), the corresponding slave system (11), and the controller (12) thus complete the STDS.  □

## 4. Speech Secure Communication

The time-delay complex Lü system signal is inherently random and is difficult to replicate. In this article, according to the characteristics of the chaotic system, an audio codec based on the time-delay complex Lü chaotic system is proposed using the designed controller, which is used to encrypt the transmission of speech signals. This kind of audio encoding body is easier to implement than other schemes in the transmission process and its recovery effect is very good, with the signal basically being completely recovered. Moreover, it is not easily destroyed in the transmission process due to the time-delay characteristics of chaotic signals [32,33].

The following complex Lü system *L1* (16) is used as the transmitter, while the complex time-delay Lü system *L2* (11) is used as the receiver.

(16) $$L1:\left\{\begin{array}{l} {\dot{u}}_{1}=a_{1}(u_{3}-u_{1})+bh_{i}\\ {\dot{u}}_{2}=a_{1}(u_{4}-u_{2})+bh_{j}\\ {\dot{u}}_{3}=a_{2}u_{3}-u_{1}u_{5}\\ {\dot{u}}_{4}=a_{2}u_{4}-u_{2}u_{5}\\ {\dot{u}}_{5}=-a_{3}u_{5}+u_{1}u_{3}+u_{2}u_{4} \end{array}\right.$$

**Figure 11:**
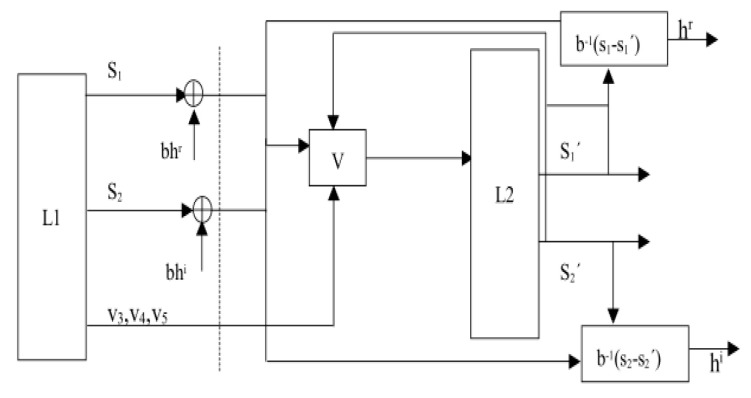
Block diagram of our communication scheme.

Figure 11 shows a block diagram of our communication scheme. At the sending end of the speech transmission, cheerful “Traveling” music was selected as the audio signal, the *L1* system was used as the encrypted voice signal, and the *L2* system was used as the decrypted voice signal. The transmitted signals were related to all parts of the master system and information signal. There is no longer a need to transmit each information signal using a separate channel. The transmitted signals are the state variables of the main system and the linear equations are encrypted to improve the security of communication. The transmission signal at the sending end is expressed as $s_{1}(t)=a_{1}u_{3}(t-\tau )-k_{1}u_{1}(t-\tau )+bh^{r}$, $s_{2}(t)=a_{1}u_{4}(t-\tau )-k_{2}u_{2}(t-\tau )+bh^{i}$, where $h^{r},h^{i}$ are the information signals and *b* is the parameter. The superscripts *r* and *i* stand for the real and imaginary parts of the complex vector, respectively. The signals at the receiving end are a linear combination of the variables of the master system and the slave system, expressed as $s_{1}^{\prime }(t)=a_{1}u_{3}(t-\tau )-k_{1}{u^{\prime }}_{1}(t-\tau )$ and $s_{2}^{\prime }(t)=a_{1}u_{4}(t-\tau )-k_{2}{u^{\prime }}_{2}(t-\tau )$, respectively. The controller (17) evolved from the STDS controller (12) is used as our speech communication controller:

(17) $$\left\{\begin{array}{l} v_{1}=s_{1}-s_{1}^{\prime }=k_{1}e_{1}+bh^{r}\\ v_{2}=s_{2}-{s^{\prime }}_{2}=k_{2}e_{2}+bh^{i}\\ v_{3}=u_{1}^{\prime }u_{5}^{\prime }-u_{1}u_{5}+k_{3}e_{3}+a_{2}(u_{3}-u_{3}(t-\tau_{1}))\\ v_{4}=u_{2}^{\prime }{u^{\prime }}_{5}-u_{2}u_{5}+k_{4}e_{4}+a_{2}(u_{4}-u_{4}(t-\tau_{1}))\\ v_{5}=a_{3}(-u_{5}+u_{5}(t-\tau_{0}))-u_{1}^{\prime }u_{3}^{\prime }-\;u_{2}^{\prime }u_{4}^{\prime }+\;u_{1}u_{3}+\;u_{2}u_{4}+k_{5}e_{5} \end{array}\right.$$

The recovered signal is $h_{g}=b^{-1}(s-s^{\prime })$ and the error is $e_{m}(t)=h(t)-h_{g}(t)$. As the speech signal has a large number of samples, it is necessary to observe several fragments, which were sufficient for completing the simulation experiment. In addition, the encrypted signal completely covers the original speech signal, such that the eavesdropper is less likely to extract the original speech.

Compared with other examples of communication systems [34,35,36,37,38,39,40], the proposed secure communication scheme based on the time-delay complex Lü system has the following advantages:

1. Due to the time lag in the transmission process, the synchronization phenomenon between the transmitter and the receiver is closer to the real situation;
2. The STDS controller based on Lyapunov’s stability design is relatively simple and has strong stability. Equipped with double time-delay, the complex Lü system is safer;
3. Encryption is performed using linear equations of state variables, which is quite different from previous encryption methods.

**Remark** **4.** *The b value is only used to adjust the signal amplitude, in order to ensure that the transmitted signal can completely cover the information signal; its magnitude can be positive or negative.*

## 5. Simulation Experiment

### 5.1. STDS Controller Simulation

**Figure 12:**
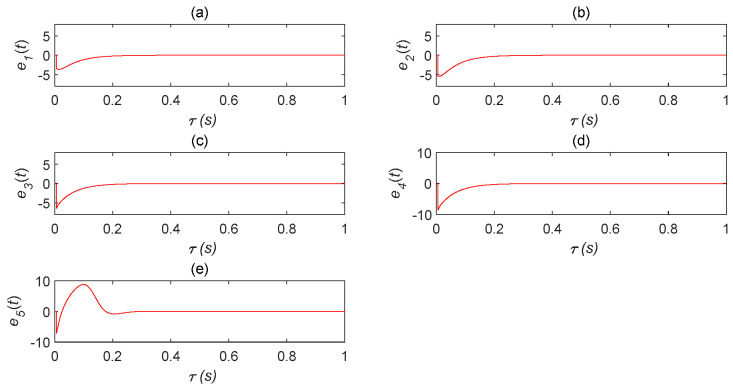
Self-time-delay synchronization error status analysis diagram. (**a**) error analysis chart of ${u^{\prime }}_{1}$ and $u_{1}$ changing with time; (**b**) error analysis chart of ${u^{\prime }}_{2}$ and $u_{2}$ changing with time; (**c**) error analysis chart of ${u^{\prime }}_{3}$ and $u_{3}$ changing with time; (**d**) error analysis chart of ${u^{\prime }}_{4}$ and $u_{4}$ changing with time; (**e**) error analysis chart of ${u^{\prime }}_{5}$ and $u_{5}$ changing with time.

The complex Lü system (2) was taken as the master system and the double time-delay complex Lü system (11) as the slave system. The initial values of the system were u(0)=[1,2,3,4,−1] and $u^{\prime }(0)=[-2,-3,-3,-4,-8]$, while the time-delays were $\tau_{0}=5,\tau_{1}=3s$. The controller of system (12) was adopted, where $k_{1}=k_{2}=-20$, $k_{3}=k_{4}=-44$, and $k_{5}=-70$. The STDS state error graph was obtained, as shown in Figure 12. The effect of this simulation was better. The error tended to be zero and the simulation results were basically consistent with the mathematical analysis, verifying the effectiveness of the controller.

**Figure 13:**
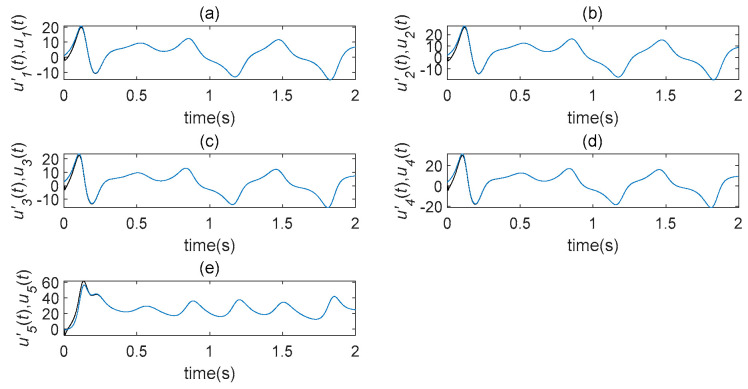
Self-time-delay synchronization state analysis diagram. (**a**) STDS state analysis diagram of ${u^{\prime }}_{1}$ and $u_{1}$ changing with time; (**b**) STDS state analysis diagram of ${u^{\prime }}_{2}$ and $u_{2}$ changing with time; (**c**) STDS state analysis diagram of ${u^{\prime }}_{3}$ and $u_{3}$ changing with time; (**d**) STDS state analysis diagram of ${u^{\prime }}_{4}$ and $u_{4}$ changing with time; (**e**) STDS state analysis diagram of ${u^{\prime }}_{5}$ and $u_{5}$ changing with time.

**Remark** **5.** *As the controller has better effectiveness, the error system is rapidly stabilized and, as shown in*Figure 13*, the waveform started to overlap with x(t) and y(t) after*τ=0.3s.

### 5.2. Speech Communication Simulation

System (16) was used as an *L1* transmitter, system (11) was used as an *L2* receiver, and system (17) was used as a controller for the MATLAB simulation. The initial values of the system were set to u(0)=[1,2,3,4,−1] and $u^{\prime }(0)=[-2,-3,-3,-4,-8]$. The system parameters were set as $a_{1}=45,$ $a_{2}=25,a_{3}=6$ and the time-delays were set as $\tau_{0}=5s,\tau_{1}=5s$. The “Travelling” music, as an audio signal, was transformed into a waveform signal in MATLAB for encryption and decryption. 

**Figure 14:**
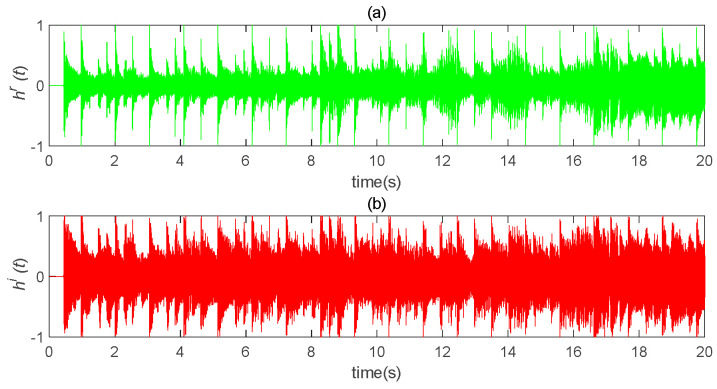
Schematic diagram of “Traveling” original waveform. (**a**) the real part of the original speech signal changes with time; (**b**) the imaginary part of the original speech signal changes with time (*b* = 300).

**Figure 15:**
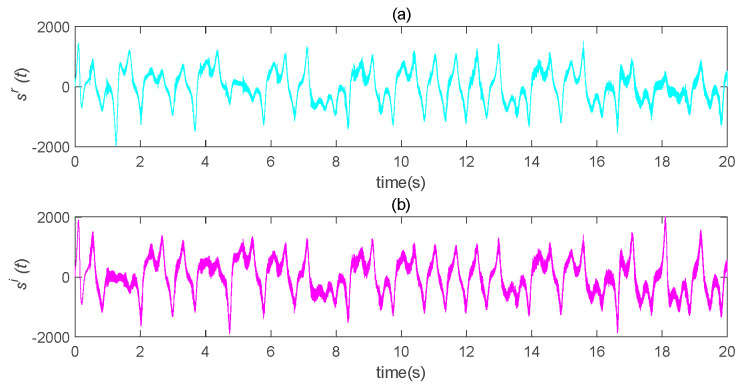
Schematic diagram of transmission encryption speech. (**a**) the real part of the encrypted speech signal changes with time; (**b**) the imaginary part of the encrypted speech signal changes with time.

**Figure 16:**
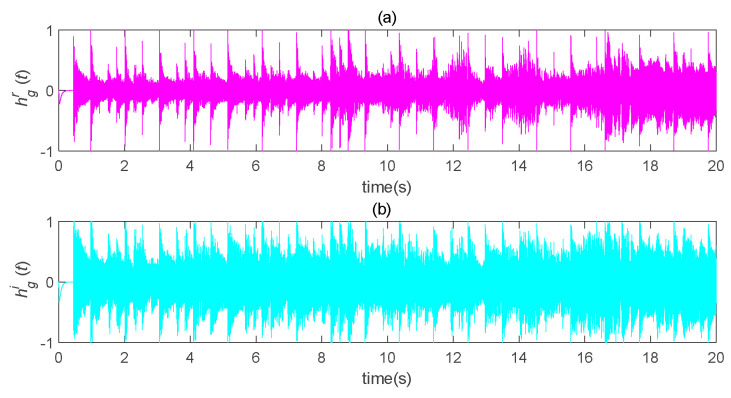
Schematic diagram of speech recovery after chaos masking. (**a**) the real part of the recovered speech signal changes with time; (**b**) the imaginary part of the recovered speech signal changes with time.

The original speech signal, h(t), is shown in Figure 14. The transmission signal, *s(t)*, is an encrypted signal which completely hid the information signal, as shown in Figure 15. The transmission signal was a combination of state variables and information signals of the master system. For some signals with larger amplitude, the *b* value could be appropriately reduced to ensure the shielding effect of chaotic signals on information signals. The recovered signal is shown in Figure 16. Compared with Figure 14, it can be seen that the original signal was recovered well.

**Figure 17:**
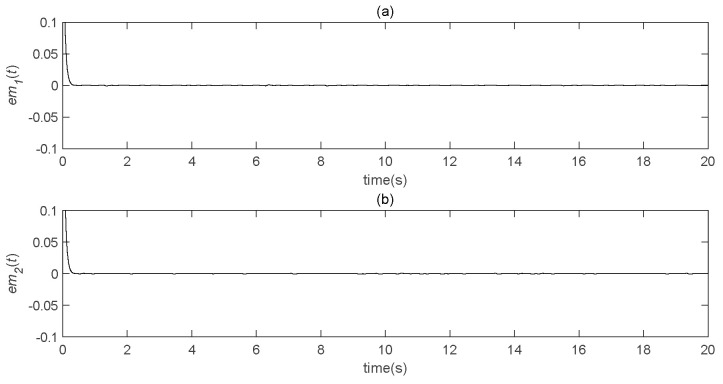
Speech transmission error analysis diagram (*b* = 300). (**a**) the real part error between the original speech signal and the restored speech signal varies with time; (**b**) the imaginary part error between the original speech signal and the restored speech signal changes with time.

It can be seen, from Figure 17, that the error rapidly tended to zero; furthermore, it can be seen from the other pictures that the effect of speech encryption communication based on chaotic concealment was very good. The figures also show that the self-delay synchronization controller could achieve speech transmission relatively well. Moreover, it is easily implemented in the actual process.

### 5.3. Effect of the Parameter b

**Figure 18:**
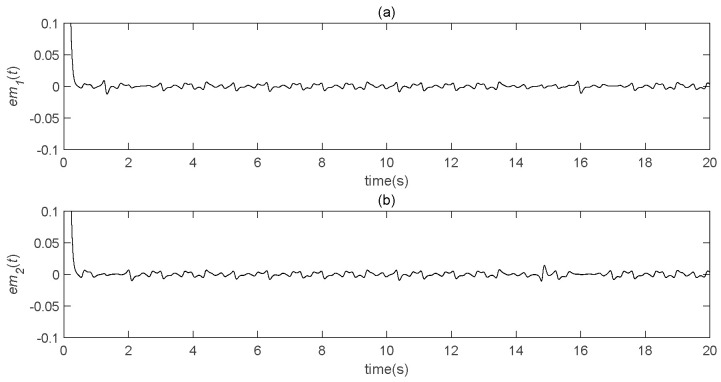
Speech transmission error analysis diagram (*b* = 20). (**a**) the real part error between the original speech signal and the restored speech signal varies with time; (**b**) the imaginary part error between the original speech signal and the restored speech signal changes with time. If you have any questions about the article, please contact us in time, we will complete it in time and do it well.

In order to verify the effect of the parameter *b*, many simulation tests were carried out. It can be seen, from Figure 18, that the recovery effect was not very good when *b* was relatively small. When the value of *b* gradually increased, the error became smaller and, when *b = 300*, the error tended to zero. Finally, when the value of *b* continued to increase, the error essentially did not change; that is, it had reached a steady state. Therefore, the best decryption effect can be obtained by changing the value of *b*.

## 6. Conclusions

In this investigation, the time-delay complex Lü system was first studied—its chaotic characteristics were analyzed by Poincare and Lyapunov analysis methods, and the chaotic attractor and Poincare cross-section were given. Then, a synchronous controller was designed to establish the STDS of the time-delay complex Lü system and strict mathematical proof was given. Next, by applying the previously designed controller, speech communications were encrypted in the form of linear equations of state variables. Finally, a simulation experiment was conducted using MATLAB simulation. The simulation results were consistent with the theoretical analysis, thus verifying the effectiveness of the controller. It was shown that the controller can restore the original voice signal well, in terms of secure speech communications.
